# Synthesis of Nanogels: Current Trends and Future Outlook

**DOI:** 10.3390/gels7020036

**Published:** 2021-03-29

**Authors:** Emanuele Mauri, Sara Maria Giannitelli, Marcella Trombetta, Alberto Rainer

**Affiliations:** 1 Department of Engineering, Università Campus Bio-Medico di Roma, via Álvaro del Portillo 21, 00128 Rome, Italy; e.mauri@unicampus.it (E.M.); s.giannitelli@unicampus.it (S.M.G.); m.trombetta@unicampus.it (M.T.); 2 Institute of Nanotechnology (NANOTEC), National Research Council, via Monteroni, 73100 Lecce, Italy

**Keywords:** nanogels, colloids, chemical crosslinking, physical crosslinking, microfluidics, lab-on-a-chip, 3D printing

## Abstract

Nanogels represent an innovative platform for tunable drug release and targeted therapy in several biomedical applications, ranging from cancer to neurological disorders. The design of these nanocarriers is a pivotal topic investigated by the researchers over the years, with the aim to optimize the procedures and provide advanced nanomaterials. Chemical reactions, physical interactions and the developments of engineered devices are the three main areas explored to overcome the shortcomings of the traditional nanofabrication approaches. This review proposes a focus on the current techniques used in nanogel design, highlighting the upgrades in physico-chemical methodologies, microfluidics and 3D printing. Polymers and biomolecules can be combined to produce ad hoc nanonetworks according to the final curative aims, preserving the criteria of biocompatibility and biodegradability. Controlled polymerization, interfacial reactions, sol-gel transition, manipulation of the fluids at the nanoscale, lab-on-a-chip technology and 3D printing are the leading strategies to lean on in the next future and offer new solutions to the critical healthcare scenarios.

## 1. Introduction

Nowadays, the design of nanocarriers plays a leading role in the formulation of advanced therapeutic treatments for several acute and chronic diseases, ranging from tumors to neurodegenerative scenarios. The manipulation of matter at the atomic and molecular levels offers different configurations of colloidal nanocarriers: organic (such as solid lipid nanoparticles, liposomes, dendrimers, polymeric nanoparticles and micelles), inorganic (carbon nanotubes, metallic and silica-derived nanostructures) and hybrid systems (combination of organic and inorganic materials) [1,2,3]. All of them have been investigated as efficient tools for the delivery of bioactive species, designing the so-called ’controlled drug delivery’ strategy, where the pivotal aim is the tunable release of the payload within the therapeutic concentration range, hence minimizing the ineffectiveness or the potential side effects of the treatment [4]. However, the relevance of the nanocarriers is not limited to this task: they also serve as platforms for diagnosis, monitoring or theranostics, in order to promote the administration of a single nanosystem for a combinatorial and multifunctional therapy [5,6]. This approach involves different routes to obtain nanovehicles with high selectivity towards target cells, and the consequent internalization and nanostability within the cytosol. Many strategies have been proposed to tune the physical, chemical and mechanical features of the nanocarriers. In particular, nanostructured gels (nanogels) have gained considerable interest and have become the subject of several studies focused on novel production methods and new areas of application.

Nanogels (NGs) can be defined as submicron-sized hydrogels, formed by physically or chemically crosslinked polymeric chains which give rise to a three-dimensional (3D) tunable porous network with a high capacity to absorb water, without actually dissolving into an aqueous medium [7]. Typically, they are characterized by spherical shape, but other configurations can occur depending on the fabrication methods: for example, micromolding techniques and photolithography allow nanogel size and shape to be controlled by modulating the surface energy and the chemical interactions among the polymers [8,9,10,11]. Moreover, NGs can be designed to have a crosslinked core-shell, a core-shell-corona or a bulk (similar to a ‘ball of wool’) structure.

The distinctive feature of these nanomaterials is their swelling behavior. The polymers used in NG synthesis absorb water giving a mostly hydrophilic nature to the final nanonetwork, capable of incorporating a great amount of water or biological fluids while maintaining its structural integrity. The phenomenon is led by the contact between the nanoscaffold and the solvent molecules: the latter interact with the polymeric surface *via* dipole-dipole interactions, London dispersion forces or hydrogen bonds, and penetrate the network. The polymer chains start to elongate, expanding the nanostructure, until the elastic retroactive force counterbalances the deformation of the system. The resulting condition is an equilibrium of stretching−shrinking, which allows NGs to uptake more than 90% *w*/*w* of aqueous solution [12]. The ability to retain water enhances the diffusion and the exchange of ions, metabolites, and biomolecules to and from the biological compartments (i.e., tissue fluids and organs), in order to maintain the biological-chemical balance with the surrounding environment: this shows good synergy between NGs and biological applications [13,14], making them highly promising biocompatible candidates. Moreover, NG swelling can be engineered to be sensitive to external stimuli, such as pH and temperature variations, ionic strength, and solvent affinity: this helps to tune NG porosity, stiffness and size, so as to achieve a controlled, triggered response at the target site [5,15,16].

Therefore, in drug delivery, NGs not only protect the payload from undesired degradation and early release, but also actively participate to the delivery process.

The smart choice of the polymers and the resulting architectural versatility enable NGs to incorporate a plethora of hydrophilic and lipophilic molecules, ranging from inorganic compounds to biomolecules such as amino acids, proteins and nucleic acids (DNA, RNA), without compromising their gel-like behavior. 

NG multifunctionality is a key property which is difficult to find in other types of nanoparticulate systems and is the result of considerable research efforts to improve and optimize NG formulations.

This review aims to give an overview on the synthetic routes commonly used to fabricate NGs for drug delivery and targeted therapies. The discussion will be focused on the description of the traditional and advanced methodologies for NG synthesis, highlighting the physico-chemical principles, the main advantages and limitations, and the most common precursors in NG design. For each synthetic strategy, applicative examples will be provided. Moreover, a summary of the main chemical reactions involved in the modification of NGs will be addressed. Indeed, the functionalization of nanogels represents a complementary approach to face the challenge of developing nanoscaffolds for selective targeting and encapsulating entities with very different physical properties within the same carrier.

## 2. Fundamental Criteria in NG Synthesis

Beyond the swelling behavior, which can be classified as a ‘superior’ property of the NGs, the main features to be considered in the synthesis of these nanomaterials are: high biocompatibility, biodegradability, colloidal stability, high surface area, high loading capacity ensuring a sustained and targeted drug delivery, and active/passive drug release thanks to the particle size and the surface properties [11]. In addition, other features that can be tuned by carefully controlling the NG synthetic routes include:

- Release of both water-soluble and oil-soluble bioactive compounds;
- Versatility in administration route (i.e., mucosal or parenteral pathway);
- Low immunogenicity and reduced NG elimination by the mononuclear phagocytic system (MPS);
- Optimization of NG permeability;
- Enhancement of the solubility of low-molecular-weight drugs;
- Reduction of the drug payload compared to standard drug administration.

NGs are typically composed of natural or synthetic polymers, or a combination thereof. However, their smart formulation can encompass the inclusion of inorganic components or the grafting of specific bio-moieties on the polymeric backbone. In the first case, NGs can work as imaging probes, incorporating a wide variety of diagnostic and contrast agents for different types of biomedical applications. These systems are usually defined as ’nanohybrid nanogels’ and are aimed at increasing the circulation half-lives of small molecules, serving as a highly convenient platform for combined delivery of therapeutic molecules [17]. In the second case, the conjugation of targeting ligands, antibodies, or peptides encourages the mechanism of NG active/passive targeting to the site of interest and the controlled release of the therapeutic payload.

Different strategies have been developed to address multiple applicative scenarios; however, they can all be traced back to the fundamental principles of chemistry and physics: interactions among the reactive groups of different molecules and physical parameters—such as viscosity, density and rheology—represent the basis and the key knowledge of NG design.

## 3. Traditional NG Synthesis

**Figure 1:**
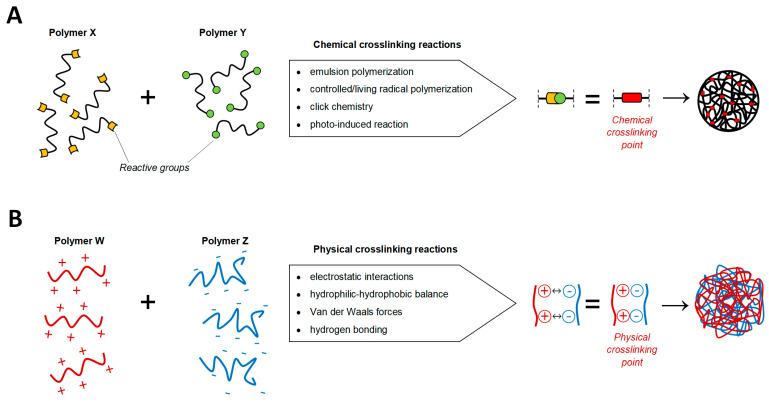
Summary of the chemical and physical crosslinking methods in nanogels (NG) design. (**A**) The formation of covalent bonds (crosslinking points, in red) between the reactive moieties (yellow and green) of polymers X and Y can be addressed through different chemical reactions exploiting the nature of the reacting groups. (**B**) The physical interactions between polymers W and Z enables the formation of a self-assembled nanoscaffold.

The methods for NG synthesis can be divided into chemical and physical ones, as shown in Figure 1. Generally, the former gives rise to nanonetworks characterized by strong covalent bonds that improve the colloidal stability under in vitro and in vivo conditions, essential for limiting the leakage of the payload induced by unwanted dissociation of the gel network. These bonds can be distinguished in:

- Cleavable linkers under specific external stimuli (pH and temperature variations);
- Stable bonds which provide the gel with the ability to retain its shape under physico-chemical stress [18,19].

Chemical crosslinking is the most developed and the most versatile strategy for NG production. On the other side, physical assembling of NGs involves a controlled aggregation mechanism led by reversible non-covalent connections (hydrophilic/hydrophobic, electrostatic, hydrogen bonding, Van der Waals, or host-guest interactions). Despite the relatively weak nanostructure due to the physical nature of the crosslinking, this process is more flexible because chemical reactions are not involved, and it occurs at mild conditions in aqueous media [20].

### 3.1. Chemical Routes

Chemical crosslinking includes the following main NG synthetic routes: emulsion polymerization, controlled/living radical polymerization, click chemistry and photo-induced crosslinking. The starting materials are low-molecular-weight monomers, polymer precursors or polymers with specific terminal or pendular reactive groups. 

#### 3.1.1. Emulsion Polymerization

**Figure 2:**
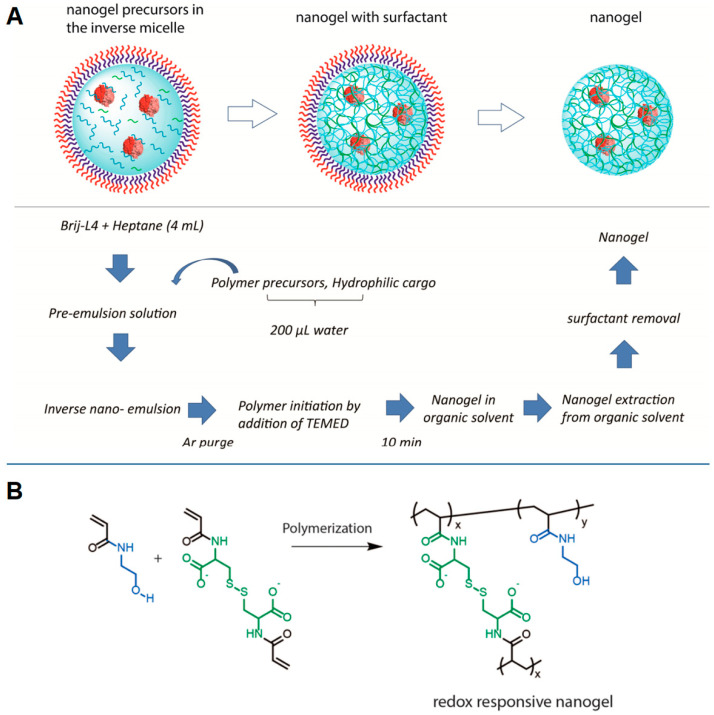
NG synthesis by inverse emulsification in the presence of Brij-L4 surfactant. (**A**) Protocol used in NG formation; (**B**) polymerization between hydroxylethylacrylamide and cystine diacrylamide giving rise to the nanonetwork. Reprinted with permission from Raghupathi et al. [22]. Copyright (2017) American Chemical Society.

Emulsion-based polymerization works through the formation of monodisperse kinetically stable droplets in a continuous phase. The rationale underlying this process is to keep the polymerization in a confined space (the droplets), whose size would affect the dimension of the final product. The dispersion of organic droplets containing the reactive monomers/polymers in aqueous solution (oil-in-water, O/W emulsion) is usually indicated as direct emulsion polymerization; whereas the aqueous droplets dispersed in an organic medium (water-in-oil, W/O emulsion) is known as inverse emulsification polymerization. NG formulation involves the use of monomers, initiators, catalysts, and crosslinking agents. Generally, the process occurs in three steps: nucleation, precursor nanoparticle growth and polymerization [21]. Two main approaches can be acknowledged. In the first one, all the reagents are dissolved in the dispersed phase and photo-initiators are preferred to activate the reaction mechanism via homolytic degradation. In the second approach, different monomers can be dissolved in the dispersed and continuous phases, respectively. A catalyst and a crosslinker are typical components of the droplets, while an initiator is added to the continuous phase. Here, thermal-initiators and reactives that are degradable via water radiolysis are commonly chosen. The resulting first radicals react with the monomers generating monomer radicals which enter the droplets via diffusion to react with the other components and form the nanonetwork: this pathway is promoted by the use of surfactants, which reduce the interface energy between the organic and the aqueous phases, or by the hydrophilic-lipophilic balance of the chemical structure of the radical itself. Raghupathi et al. [22] proposed the design of redox-responsive NGs through inverse emulsification method using hydroxylethylacrylamide and cysteine diacrylamide with the surfactant Brij-L4; the resulting nanoscaffolds were intended to encapsulate proteins as a hydrophilic cargo and to tune their release under specific stimulus triggers (Figure 2). Peres and coworkers [23] prepared glutamic acid-based NGs using methylenebis(acrylamide) as a crosslinker and sodium dodecyl sulfate (SDS) as a surfactant, and they demonstrated the effect of NG swelling behavior under pH variations in a drug delivery application.

Surfactants are used to improve the formation and stability of nanodroplets, modulating their dimension and giving nanogels with diameters less than 150 nm [24,25,26]. The resulting nano-emulsion has a high surface area and allows active components to penetrate easily and faster in the dispersed phase. The concentration of surfactant can also affect the polymerization reaction: when it exceeds the critical micellar concentration (CMC), the arrangement of surfactant at the organic-water interphase results in the formation of micelles encapsulating monomers. The consequent addition of the initiator to the emulsion gives rise to the polymerization process only after diffusing within the micelles, which therefore act as nanoreactors. Both hydrophobic and hydrophilic precursors can be used to form NGs, since the emulsion system guarantees their solubility and enhances their interfacial permeability. However, limitations of this method are related to the high amount of surfactant required and to the difficulties in achieving the complete purification of the obtained NGs. Indeed, the removal of surfactant may result in additional waste-water treatments, increasing the process costs, or in undesired contaminants in the final product. In particular, the use of non-amphiphilic polymers as nanogel precursors requires the addition of surfactants to form the nanoparticulate, and their subsequent removal during the process [27].

For these reasons, in the last years, many attempts have been devoted to developing surfactant-free emulsion polymerization (SFEP) methods. Some strategies are based on W/O emulsions, modulating the stability of the system through the volumetric ratio of the two phases. For example, Cheng and coworkers [28] have synthetized redox-responsive nanogels for drug delivery using an organic solution of PEGylated poly(amido amine) functionalized with disulfide bonds and an aqueous solution containing the drug: their mixing (through ultrasonication, shaking, or homogenization) generated a spontaneous stable W/O emulsion, where the polymer reduced the interfacial tension and filled up the water droplet. The crosslinked polymer network was generated via the intermolecular disulfide exchange reaction in the aqueous phase. Otherwise, the organic phase could be composed by the monomers: Ashrafizadeh et al. [29] proposed the design of amphiphilic pH-responsive NGs using a mixture of acrylate monomers and a dimethacrylate cross-linker combined with an aqueous solution of initiatior to activate the emulsion polymerization. Furthermore, another innovative approach has been discussed by Wang and collaborators [30], who have prepared NGs by emulsion-free photopolymerization: they combined the self-emulsification of poly(ethylene glycol) diacrylate monomer and the small irradiation region of a low-cost semiconductor laser to achieve a spatiotemporally controllable photopolymerization. 

Another technique, which represents a milestone in NG design, is the emulsification-solvent evaporation [31,32]. In this case, the approach is preferentially based on the covalent crosslinking of preformed polymer chains, instead of monomers polymerization, to provide excellent opportunities for producing nanogels with tunable pore size [33,34]. Vinogradov, who share with Akiyoshi’s group the development of the first nanogels, focused on this method to formulate a wide range of nanonetworks. In this procedure, an activated polymer is dissolved in an organic phase and the other one in water; the addition, under vigorous stirring or sonication, of the former to the latter gives rise to a O/W emulsion, followed by the evaporation in vacuum of the organic solvent and the progressive maturation of the NGs in the aqueous phase. The formation of covalent bonds between the polymers starts at the interface and continues in water. In this scenario, the presence of reactive groups is essential to generate the nanoparticles: these moieties can be existing in the polymer backbone or can be grafted to it by post-polymerization functionalization, and they shall maintain their reactivity in the reaction system. Equally, the choice of the organic solvent is important: it should be poorly soluble in the continuous phase, have high volatility and low boiling point, be able to dissolve the polymer and preferably hold reduced toxicity. The main solvents used are: dichloromethane, ethyl acetate and ethyl formate [31].

Literature reports several application examples of this technique. Vinogradov and coworkers synthetized cationic NGs composed of imidazole-activated PEG and branched PEI for antiviral drug delivery against HIV infection in the brain [35,36,37] and amphiphilic cationic NGs based on cholesterol-ε-polylysine to deliver therapeutic nucleoside reverse transcriptase inhibitors (NRTI) in the central nervous system, limiting the neurotoxicity [38]. A similar protocol was used by Mauri and coworkers to design NGs made of PEG and linear PEI for drug delivery in astrocytes [39] and microglia [40] as treatments for spinal cord injury. Li and coworkers used carboxymethyl chitosan in the fabrication of NGs for doxorubicin release in tumor-like multicellular spheroids [41].

#### 3.1.2. Controlled/Living Radical Polymerization

Another polymerization technique of major interest is the controlled/living radical polymerization (CLRP). Since 1990s, CLRP has been explored to synthetize either crosslinked particles or gels with well-defined polymer molecular weight through the addition of crosslinking agents [42]. It can be conducted under simple polymerization conditions and a wide range of chemical reactive groups and solvents, including protic media such as water, can be used to achieve a high control on the polymerization process, almost as good as that of living anionic polymerization, and to maintain the characteristic tolerance and flexibility of a free radical process [43]. The use of a wide range of vinylic monomers allows the production of nanogels with different composition, dimensions, and architectures, including core–shell and hollow configurations. Moreover, functional initiators or macroinitiators ensure the grafting of specific moieties in the NG inner core or surface, facilitating multivalent bioconjugation [7,44]. Similar to conventional radical polymerization, CLRP mechanism is based on four elementary steps: initiation, propagation, transfer chain reaction and termination. The essential differences lie in:

- The initiation phase, which is faster (compared to the corresponding propagation and termination reactions) than in standard radical polymerization reactions;
- The generation of a dynamic equilibrium between a low concentration of radicals and a large amount of dormant reactivatable species in the propagation phase;
- A considerably slower global kinetics than conventional radical polymerization.

As discussed in detail by Sanson et al. [44], these features promote an ideal condition of an almost constant number of chains throughout the polymerization, which are initiated nearly at the same time and having the same growth rate: this ensures an inner control over molar mass distribution and architecture. Furthermore, the slow CLRP mechanism allows a gradual reactivation of the dormant chains, permitting them to diffuse and be homogeneously distributed. As a result, the crosslinking points forming the NGs are more evenly distributed within the nanonetwork, which features a locally branched polymeric structure, more regular than the standard radical approaches (where the formation of dense/nodular crosslinking domains occurs, producing a heterogeneous nanostructure) [45]. The success of CLRP in NG design is related to the control of the network formation: the nanoscaffolds can be modulated in size and molar mass. Depending on the concentration of monomers and crosslinking agent, the NG building blocks can have different ranges of molar mass and branched structure, tuning the final morphology and structural composition (such as the porosity) of the nanogels and thus achieving a variety of architectures [46,47,48]. As reported in Table 1, the pivotal strategies of CLRP are: nitroxide-mediated polymerization (NMP) [49], atom transfer radical polymerization (ATRP) [50], reversible addition–fragmentation chain transfer polymerization (RAFT) [51], iodine-mediated polymerization (RITP) [52], and polymerization/macromolecular design via the interchange of xanthates (MADIX) [53]. 

**Figure 3:**
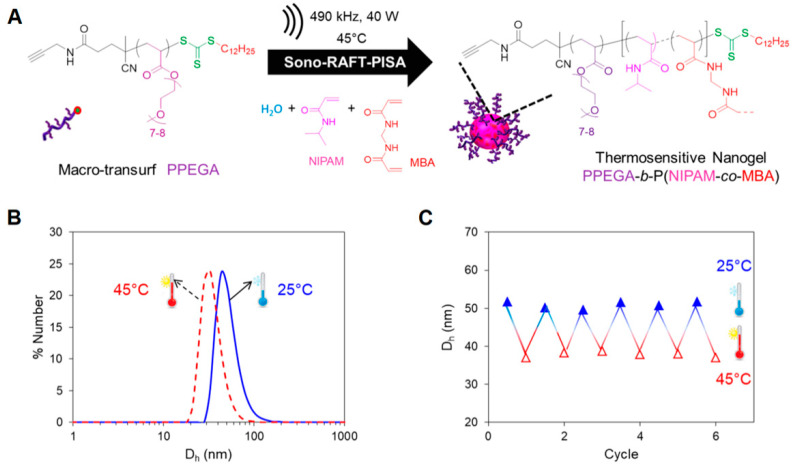
Sonochemically induced reversible addition–fragmentation chain transfer polymerization (RAFT) polymerization to design thermosensitive NGs. (**A**) Synthesis of (PPEGA-b-PNIPAM) NGs; (**B**,**C**) Dynamic Light Scattering analysis (DLS) of the obtained NGs: the size (hydrodynamic diameter, D_h_) of the nanonetwork differs in heating (45 °C) and cooling (25 °C) experiments, demonstrating the thermosensitive behavior. Adapted with permission from Piogè et al. [61]. Copyright (2018) American Chemical Society.

Recently, Dinari and coworkers [58] have applied ATRP in fabricating dual responsive lignin-based nanogels for controlled release of curcumin, whereas in the work of Lou et al. [59] a core-shell NGs with thermo- and redox-sensitive properties was developed via ATRP using poly-N-isopropylacrylamide and zwitterionic copolymer blocks containing poly(sulfobetaine methacrylate) and a lactose motif of poly(2-lactobionamidoethyl methacrylamide). The resulting NGs demonstrated selective targeting towards hepatoma receptors and were used for doxorubicin delivery in hepatic cells (HepG2). On the other side, as shown in Figure 3, Piogé and coworkers [61] have proposed the design of Low Critical Solution Temperature (LCST)-type thermosensitive NGs made of poly[poly(ethylene glycol) methyl ether acrylate] and poly-N-isopropylacrylamide (PPEGA-b-PNIPAM), combining RAFT mechanism and high-frequency ultrasound. Poly et al. [68] synthetized poly(vinyl acetate) NGs by radical crosslinking controlled by xanthate, which ensured a high yield and a homogeneous structure in the nanonetwork. 

#### 3.1.3. Click Chemistry

**Figure 4:**
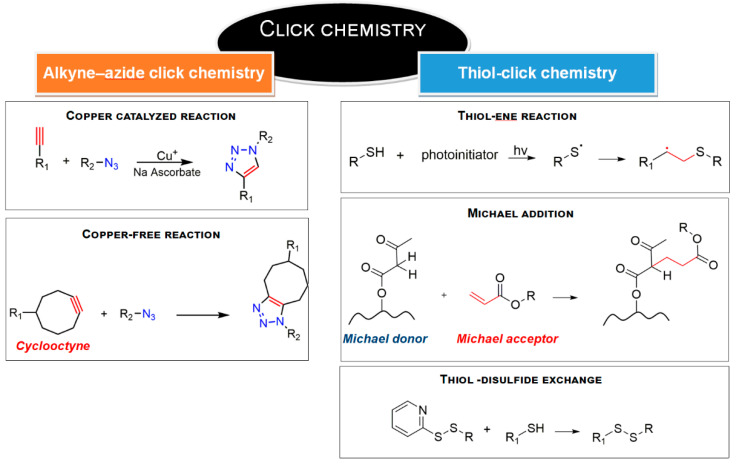
Click chemistry reactions involved in NG synthesis.

Emerging NG designs have also taken advantage of click chemistry approaches [70,71]. This strategy ensures short reaction time, high yield and purity, regiospecificity, versatility and aqueous reaction conditions. The mechanism involves the presence of azide and alkyne groups on the building blocks to form a stable conjugate (triazole, Figure 4). Copper-catalyzed and copper-free strain-promoted azide−alkyne cycloadditions are the reference reactions.

As discussed by Sharpless [72], the reaction is generally not affected by steric factors and substituted primary, secondary, tertiary, aromatic azides can readily participate in this transformation with alkyne-derived components. A potential limitation of this approach is related to starting materials missing of the reactive groups: however, azide and triple bond can be easily grafted on polymeric backbones (for example, via nucleophilic substitution [73,74]) and the resulting compounds are extremely stable in standard conditions [75]. Indeed, high tolerance to oxygen, aqueous and common organic solvents, synthesis conditions, biological molecules, and pH has been demonstrated [76]. Zhang and coworkers [77] synthetized crosslinked prodrug nanogels using polyethylene glycol (PEG) modified with polypropargyl glutamate and doxorubicin functionalized azide: the click reaction led to 3D nanoscaffolds feasible for selective intracellular drug delivery in tumors, such as human breast adenocarcinoma and cervical cancer. Additionally, the work of Ding et al. [78] showed the conjugation between dibenzocyclooctyl-modified DNA and azide-modified polycaprolactone (PCL) by a copper-free click reaction: the result was a nucleic acid-based NG, designed for gene delivery and antitumor therapy.

Another reaction that might be classified as a ‘click chemistry’ approach is the thiol-click chemistry [79,80] (Figure 4). Mainly, it includes thiol−alkene and thiol−alkyne reactions, nucleophilic Michael addition and disulfide exchange; pH-sensitive NGs can be produced with this strategy: for example, the polymerization between methoxy polyethyleneglycol acrylate (mPEGA), pentaerythritol tetra(3-mercaptopropionate) (PT) and ortho ester diacrylamide (OEAM) was investigated by Wang and collaborators [81] to obtain NGs characterized by an acid-cleavable network enable to perform pH-triggered drug release in intracellular acid environment. Zhang and coworkers [82] used thiol-alkene reaction to develop highly structured dendritic NGs through the divergent growth approach from bifunctional or monofunctional PEG and propionic acid-derivative monomers, for the successful delivery of chemotherapeutics in 3D pancreatic spheroids tumor model.

#### 3.1.4. Photo-Induced Crosslinking

Photo-crosslinking represents an alternative approach to the above-described polymerization-based design of NGs. It is defined as a ‘clean method’, because no crosslinking agents and/or catalysts are necessary, and no by-products are formed [83,84]. Polymers functionalized with photo-activatable or dimerizable groups are used to give rise to a stable NG architecture, produced through the formation of covalent bonds. The reaction involves the presence of a photoinitiator, which is converted into reactive radical species via photolysis or light-induced cleavage. The rate of formation of the initial radicals affects the spatial distribution of the covalent crosslinking and, consequently, the microscopic and macroscopic characteristics of the formed nanonetwork (i.e., swelling behavior, stability and surface area). Together with the polymer concentration, the intensity of the incident light, the type and concentration of the photoinitiator, the quantum yield and the number of radicals generated per photolysis event are the main parameters affecting photo-crosslinking reaction [85]. However, although the photo-induced crosslinking is highly efficient and characterized by short reaction time, the initiator may induce cytotoxicity in the produced nanoscaffolds: for this reason, the choice of photoinitiator is intended to cause the minimal toxicity [18]. For example, Irgacure 2959 is well tolerated by many cell types and suitable for optimal NG design [86].

In the last years, Chen and collaborators [87] synthetized pH-degradable polyvinyl alcohol (PVA)-based NGs through photo-crosslinking of thermal-preinduced nanoaggregates. The synthesis was conducted in aqueous solution, using Irgacure as initiator and under UV exposure in an inert gas atmosphere. Paclitaxel was encapsulated and its release kinetics was investigated in acidic and physiological conditions: the responsive behavior of NGs ensured a pH-triggered drug release under intracellular acidic conditions, highlighting this system as a promising nanoplatform for delivery of active molecules in tumoral scenarios. Kim and coworkers [88] obtained gelatin methacrylate NGs by UV-induced nanonetwork formation: the resulting system was applied as nanocarrier for transdermal delivery of hydrophilic macromolecules.

Moreover, photo-activation principles are also applied in the photo-mediated redox crosslinking. The nanonetwork is obtained by polymers carrying phenol moieties, that can be activated, in the presence of a photosensitizer, via photooxidation, generating a subsequent radical coupling between the reactive groups and the final crosslinking [89]. Regarding the photosensitizer molecules, their choice falls on dyes or additives that absorb light and promote a transition towards an excited state, which ensures their capability of oxidizing the reactive groups of interest. They also have high quantum yield and sufficient stability to catalyze the photooxidation [90]. 

### 3.2. Physical Routes

The formation of 3D nanoscaffolds can be also reached by polymer-polymer and polymer-biomolecule physical interactions. In this case, supramolecular crosslinking occurs, due to the generation of nanoaggregates via self-assembly. Ionic, hydrophilic-hydrophobic, Van der Waals and hydrogen bonds are the driving forces, without the addition of crosslinking agents that might cause undesired interactions during NG formation and the encapsulation of bioactives, affecting the performance of drug loading. Compared to chemical strategies, NG engineering through physical crosslinking involves mild synthesis conditions, mostly in water, limited adverse toxic effects, and NG size can be tuned by regulating the polymers concentration and the experimental conditions, such as ionic strength, temperature, and pH. 

Temperature-responsive and pH-responsive monomers or polymers can be used to address the self-assembly of nanonetworks and to control the drug release performance. These starting materials can also modulate the NG structure, shape and size according to the external stimuli. Thermo-sensitive compounds undergo a reversible phase transition in aqueous media in response to temperature variations; in particular, thermo-sensitive polymers exhibit a critical solution behavior according to the temperature-dependent polymer-polymer and polymer-solvent interactions. This defines the critical solution temperature (CST) as the key parameter that manages the nanonetwork assembly: it represents the value at which the polymer, dissolved in the solvent, generates a second separated phase where the chains tend to form a compacted coil state [91]. NGs can be formed by materials characterized by a low critical solution temperature (LCST) or an upper critical solution temperature (UCST): in the first case, the generation of the nanoscaffolds occurs at values above LCST; whereas in the second case, NGs are synthetized at temperatures below UCST. In addition, both these temperatures can be tuned through the functionalization of the polymer backbones with specific moieties: LCST can decrease by grafting pendular hydrophobic moieties [6], whereas the UCST phase transition can be affected by changing the polarity of the polymers and their capability of engaging in hydrogen and electrostatic interactions [92]. Such tunability allows the synthesis of NGs in the desired temperature window and the generation of NGs with specific operative temperatures for applications including hyperthermia and theranostics. Thermo-sensitive polymers can bear amine, polyether, vinyl ether, acrylate, and hydrophobic groups. For example, Paradossi’s group has widely discussed the use of poly(N-isopropylacrylamide) (PNIPAM) to develop nanoscaffolds with temperature-responsive properties, which enable the active targeting of tumor cells and sustained drug release [93,94]. Sliwa et al. [95] have synthetized a thermo-responsive nanogel by PNIPAM and vinylimidazole, characterized by size variations with changing temperature or pH, which modulates the release of the dye Orange II. Instead, Ohshio et al. [96] exploited the UCST of a random copolymer with lateral ureido groups and primary amines combined with poly(2-methacryloyloxyethyl phosphorylcholine (PMPC) to fabricate NGs, which encapsulated hydrophobic fluorescence probes and bovine serum albumin below the UCST and released them, in a controlled manner, above the UCST.

On the other hand, pH-sensitive components possess ionizable groups which undergo protonation/deprotonation upon external pH changes. As a result, new local interactions occur among the monomers and polymers encouraging their spatial reorganization and assembling into an ordered pattern. This mechanism is driven by weak acidic or basic moieties that either accept or release protons in response to a change in the environmental pH. Polymers having carboxyl, amine, pyridine, sulfonic or phosphate groups are typically described as pH-responsive polymers because the ionization of these moieties affects the surface activity, the chain conformation and the solubility, and results in a different structural architecture of the final NGs [97]. Acrylate and methacrylate derivatives are commonly used to design pH-responsive nanoscaffolds. For example, methacrylic acid (MAA) and methyl acrylate (MA) were reported to form NGs [98] in which the pH sensitivity is correlated to their carboxyl groups: at basic pH, these groups are deprotonated and the mutual Coulombic repulsions induce NG swelling; on the other hand, at lower pH levels, the carboxyl moieties are not ionized, leading to the shrinking of the nanonetwork. Additionally, natural polymers, including chitosan, alginate, hyaluronic acid, carboxymethyl cellulose and gelatin, are used to produce NGs with swelling-deswelling variation and designed for precisely controlled drug release with the external stimulus. Indeed, exploiting this behavior, it is possible to regulate the delivery and release of drugs in a wide range of tumoral scenarios according to the pH gradients between the tumor microenvironment and the normal physiological environment [99]. 

**Figure 5:**
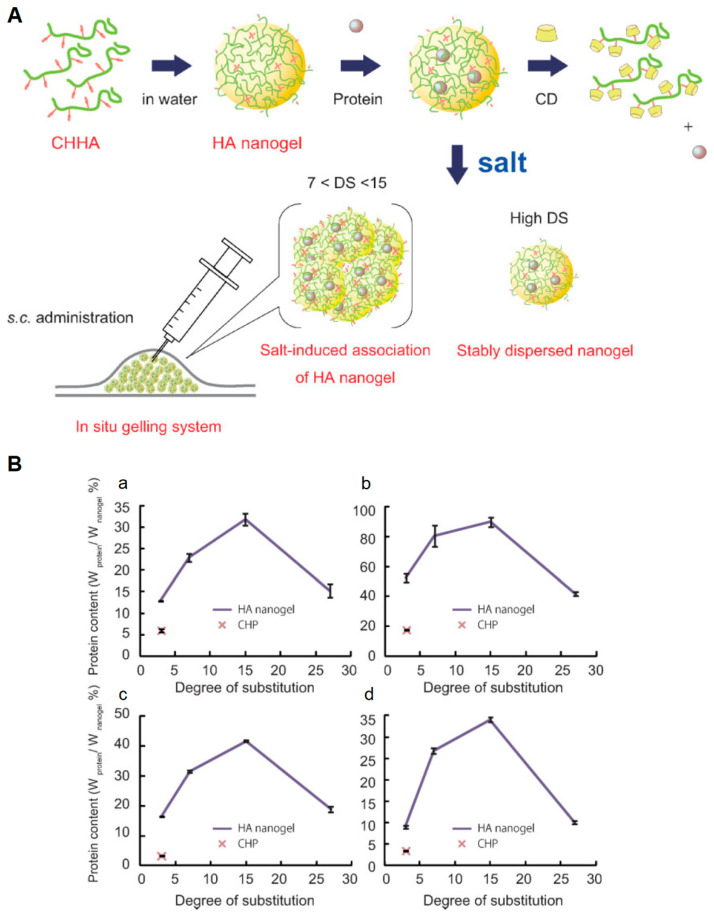
Self-assembling of hyaluronic acid (HA) NGs functionalized with cholesteryl groups. (**A**) Scheme of nanoscaffold design, protein loading and administration. (**B**) Protein-hosting capacity in the proposed NGs, using different payloads: rhGH (**a**), EPO (**b**), lysozyme (**c**) and exendin-4 (**d**). Adapted with permission from Nakai et al. [105]. Copyright (2012) WILEY-VCH Verlag GmbH and Co. KGaA.

However, physically crosslinked nanosystems possess lower mechanical strength and are more easily degradable than their covalently crosslinked counterparts: as a result, they are more sensitive to the sol−gel transition caused by environmental stimuli changes [20] and their application in harsh in vivo conditions, such as in blood circulation, is questioned [18,100]. Candidate materials for this approach are chemical structures presenting a hydrophilic framework and several grafted hydrophobic moieties, or protonatable groups: examples are gelatin, cholesterol, polysaccharides and pollulans [101,102,103,104]. For example, Nakai and coworkers [105] designed anionic NGs for protein delivery exploiting the self-assembly of cholesteryl groups grafted on hyaluronic acid (HA, Figure 5). Moshe et al. [106] modified PVA with hydrophobic moieties (isopropylacrylamide), obtaining an amphiphilic polymer with self-assembling properties, which gave rise to 3D nanonetworks. The further functionalization with boric acid domains improved NG stability, promoting their use in a sprayable form for mucosal tissue applications. 

### 3.3. Other Functionalization Strategies

To enhance the performance of NGs in drug delivery and to elicit specific therapeutic responses, the grafting of specific groups, including biomolecules, such as peptides, proteins, and growth factors represents a key approach. It can be performed through the orthogonal functionalization of the starting materials using different techniques, which require the presence of specific chemical moieties. In particular, the functionalization is usually addressed in the following ways:

- Click chemistry. The same chemical groups discussed in the previous section for NG formation can be applied to link bio-functionalities to the nanocarriers.
- Activation of esters to form amide bonds, under mild conditions. Amides are versatile linkages, characterized by unique stability towards extreme chemical environments.
- Isocyanate-based chemistry, also through modifications with alcohols, amines, and thiols. The methodology ensures high yields and stability; however, some limitations occur in the biological applications due to toxicity issues and to the sensitivity of the isocyanate to moisture.
- Imine and oxime linkages. They ensure bond reversibility due to the imine equilibrium and potential oxime hydrolysis under aqueous acidic conditions; this approach can be used to design NG systems where the release of biomolecules is tuned by hydrolysis activation.
- Ring-opening reaction. It represents a very versatile method to graft desired heteroatoms on the polymer backbone.

A thorough description of these reactions is reported by Blasco et al. [107] and Mauri et al. [12].

## 4. Advanced Fabrication Technologies

Associated to the need of having specific functional moieties for tunable drug release and targeted therapy, one major challenge is the control on NG size distribution coupled to process scalability. Conventional fabrication methods can result in extensive NG polydispersity and practical difficulties to modulate the physicochemical properties of the nanocarriers, unless the polymeric material is changed. In this context, microfluidics offers high-controllable and large-scale production yields, opening new scenarios to advanced NG design [108]. Moreover, the integration with 3D printing technologies can offer additional benefits to the production of 3D structured NGs for biomedical and pharmaceutical applications [109].

### 4.1. Microfluidic-Assisted Fabrication

Microfluidics has emerged as an innovative and advantageous approach to manipulate small amounts of reagents with accurate control on mixing and physical processes at the microscale. The advantages offered by microfluidics include miniaturization, minimized reagent consumption, decreased reaction time and enhanced process accuracy and efficiency [110]. In this framework, microfluidic technology has allowed the synthesis of a wide range of micro- and nanoproducts, including NGs, with a superior control over the product yield and the requested physico-chemical properties [110]. 

Indeed, by controlling the microfluidic conditions (including chip design, fluid rheology, and flow rates), it is possible to customize the nanosystem size, polydispersity, surface properties, payload delivery, and release profile [111]. However, due to the complexity of fluid dynamic processes, costly iterative approaches are often required to achieve the desired performance. Numerical investigations have been carried out and experimentally validated on several microfluidic devices to gain a predictive understanding of the most influential design parameters [112,113]. A significant contribution to the field has been made by Lashkaripour et al. [114], who developed an open-source tool (DAFD: Design Automation of Fluid Dynamics) that leverages machine learning to predict the parameters (e.g., droplet diameter and production rate) of flow-focusing droplet generators. Although the initial version supports a limited set of fluids, this tool has the potential to enable application-specific design optimization facilitating the adoption of functional microfluidic platforms without demanding expertise and resources.

Furthermore, since microfluidic devices often run with continuous flows, they supply high-throughput production of nanonetworks with the same quality over time, paving the way to industrial scale up [115,116]. All these benefits currently promote microfluidics as a robust approach for the synthesis of size-controlled NGs as well as for the encapsulation of antibodies, cells, or proteins with potential outcomes in the landscape of drug delivery and cell therapies [117].

#### 4.1.1. Materials for Microfluidic Devices

Besides structural and operating parameters (e.g., channel dimensions, number of inlets), the choice of the material for the fabrication of the microfluidic device is a fundamental crossroad [118]. Indeed, each material has its own features regarding processability, cost, capacity to withstand high pressures and flow rates, and compatibility with organic solvents.

**Figure 6:**
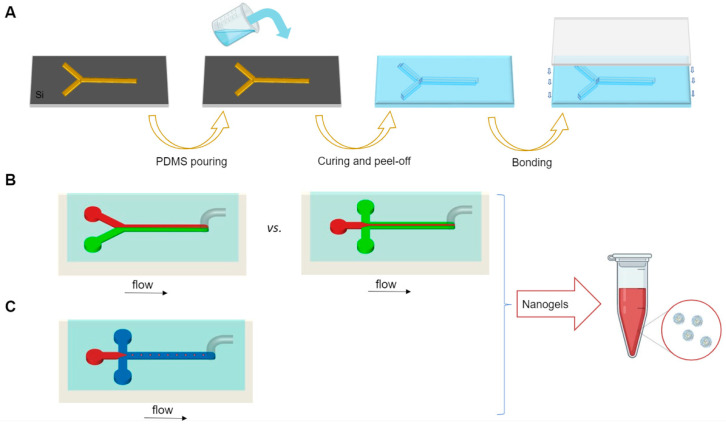
Microfluidics-assisted NG production. (**A**) Polydimethylsiloxane (PDMS) replica molding; (**B**,**C**) microfabricated microfluidic platforms: schematic of a Y-shaped microfluidic mixer (B, left), cross-shaped planar flow focusing mixer (**B**, right) and droplet microfluidic platform (**C**).

Polydimethylsiloxane (PDMS) and poly(methyl methacrylate) (PMMA) are among the most commonly used polymers for microfluidic devices. PDMS devices are usually obtained by soft-lithography [119]. In a standard soft-lithography process, PDMS replicas are obtained from lithographic masters. Although 3D printing can be alternatively used to manufacture thermoplastic masters for PDMS devices, the printing resolution is still not enough to justify a shift from traditional lithographic techniques [120,121]. The PDMS replica is then bonded to a glass substrate to seal the channels of the microfluidic device (Figure 6A). Alternatively, the obtained replica can be used as a stamp for the hot embossing of fluoropolymers [122]. Although PDMS can be easily tailored to a variety of geometries, ranging from 3D channel structures to multicompartment systems [123,124], its main disadvantage is the susceptibility to swelling when exposed to many common organic solvents, such as acetone [125]. Furthermore, the low elastic modulus of PDMS represents a shortcoming for applications requiring high pressure and high flow rate, because these parameters significantly alter the channel geometry.

Alternative polymers for microfluidic applications include polytetrafluoroethylene (PTFE) and cyclic olefin copolymer (COC), which are microstructured by hot-embossing techniques [126,127]. In addition, microfluidic devices can be also prepared by laser direct-writing of thermoplastic materials (for example, polystyrene [128] and polycarbonate [129]) at low cost and fast speed.

Although less used, glass capillary microfluidic devices have also received considerable attention. Glass capillaries can tolerate a wider range of solvents compared to their lithographically fabricated PDMS counterparts and have excellent resistance to high pressures and flow rates. Moreover, their wettability can be easily modified into hydrophobic or hydrophilic by simple chemical reactions with an appropriate surface modifier [130]. However, a laborious and technically challenging fabrication procedure often hampers the use of these devices [131].

#### 4.1.2. Microfluidic Mixing

The main applications of microfluidics for NG synthesis are focused on the nanoprecipitation and self-assembly principles. These approaches are based on the fast mixing of a solution containing the NG building blocks (polymers and biomolecules) with a miscible non-solvent of the polymers, and their use is encouraged by the straightforward procedures and their data reproducibility [132]. Several microfluidic platforms have been developed to produce nanosystems with precisely controlled diameters: the precipitation or the quick assembly of nanonetwork precursors result in smaller particle size and narrower size distribution, in comparison to the conventional methods [133,134].

In its simpler version, passive mixing has been implemented using Y- and T-shaped microfluidic schemes, which consist of two inlets and one outlet (Figure 6B). In this approach, the mixing primarily occurs at the interface of the two parallel streams flowing alongside and strongly depends on the diffusion rate [116]. To further increase the mixing, a variety of barriers to generate a chaotic flow of the molecules in the mixing channel have been added, as shown in the herringbone mixer [135] and Tesla structured channel [136]. 

In addition to these setups, hydrodynamic flow focusing (HFF) systems are among the most recognized continuous flow mixing techniques [137]. A 2D HFF mixer usually consists of three inlet microchannels and one central outlet channel organized as such that two outer fluid flows of non-solvent (continuous phases) horizontally compress the central fluid (dispersed phase) containing the nanoparticle precursors (Figure 6B). The squeezing of the core solution by the side sheath flows results in shorter mixing time leading to rapid nanoparticles production [111]. Besides the 2D-HFF devices, where the central flow is only focused on the horizontal plane, 3D-HFF have been developed to further improve the size uniformity of the synthesized nanomaterials [138]. 

Alginate NGs were successfully synthesized by Bazban-Shotorbani et al. [108] using HFF procedure in a PDMS microfluidic platform. Alginate aqueous solution was used as the core flow and was hydrodynamically focused by the lateral CaCl_2_ streams into a narrowly focused stream; hence, the polymer chains were crosslinked by calcium ions to form the alginate-based nanonetwork. Considering the anionic polyelectrolytic nature of the alginate chains, NGs were formed through a controlled diffusion-mediated mass transfer of Ca^2+^ ions into the focused polymer solution stream. The key parameter in determining NG size is the flow ratio of inlet sheath and core flows, which in turn affects the mixing time. The fine tuning of process parameters has led the production of smaller and more monodisperse nanostructures than the corresponding counterparts obtainable by the standard bulk synthesis. Furthermore, a correlation between on-chip mixing time and the average pore size of the synthesized alginate NGs was also demonstrated. In particular, a higher flow ratio between core and sheath fluid resulted in smaller and more compact nanonetwork which in turn led to higher encapsulation efficiency and slower and more controlled drug release. Indeed, NGs synthesized by the conventional bulk methods usually have a burst release and low encapsulation efficiency due to their large pore size. On the base of these findings, Mahmoudi and coworkers [139] investigated the effect of TGF-β3-loaded alginate NGs obtained through a co-flow microfluidic system on the differentiation of mesenchymal stem cells (MSCs) demonstrating their superior performance in terms of TGF-β3 release and better chondrogenic differentiation respect to the bulk synthesized nanoscaffolds. 

Although alginate has gained far more attention given its unique features such as ease of gelation and biocompatibility, to date, various kinds of polymeric NGs have been obtained through microfluidic-based approaches. Among the most notable examples, Agnello and collaborators [140] employed hyaluronic acid (HA) derivatives, functionalized with octadecyl and ethylenediamine moieties to produce nanostructures by modulating its self-assembly into a split-and-recombine micromixer. The peculiar property of the obtained HA-based specimens lies in the responsive behavior to the ionic strength, which can be exploited to produce a controlled coacervation of the polymer when mixed with aqueous salt solutions. The resulting NG size can be finely controlled in the range 150–400 nm by regulating the flow ratio. Furthermore, thanks to the very mild conditions used for their production, these nanomaterials have been used for the encapsulation of Imatinib, a FDA-approved drug to treat cell proliferation in leukemia [141]. Antiangiogenic potential of Imatinib-loaded NGs was also evaluated in vitro by using human retinal pigment epithelial cells and human umbilical vein endothelial cells.

The key concepts of controlled drug release and targeted cellular uptake have recently been addressed by the work of Elkassih et al. [142], where a fully degradable disulfide crosslinked nanogel was synthesized by oxidative radical polymerization of 2,2’-(ethylenedioxy)diethanethiol (EDDET) with different crosslinking agents. A commercial microfluidic mixing platform with herringbone rapid mixing features was used in the presence of a non-ionic Pluronic surfactant to ensure NG stability. Considering the poly(EDDET) backbone and the crosslinking junctions entirely characterized by disulfide bonds, these NGs were able to degrade intracellularly in response to redox potential through thiol-disulfide exchange reactions. Other examples of redox-responsive nanocarriers have been recently reviewed by Mi et al. [143] and successfully applied to target drugs in specific human pathological conditions, such as cancer cells characterized by remarkable higher levels of glutathione.

#### 4.1.3. Droplet-Based Microfluidics

Droplet-based microfluidics is an important subcategory of the microfluidic technologies, which manipulates immiscible/partially miscible phases to generate droplets. The droplet phase is termed as dispersed phase, while the medium phase is identified as continuous phase. Droplet generation occurs when the discrete phase intersects with an immiscible continuous phase which forces it to break into the bulk fluid stream [125] (Figure 6C). The generated droplets can then be subjected to an external trigger, such as UV light or ionic environment, to induce their crosslinking. This droplet-based microfluidic method can also allow the simultaneous loading of cells, drugs or growth factors by simply adding them into the pre-gel solution, before injection into the inner stream channel for droplet formation [144]. 

Conventional droplet-based microfluidics has been widely used to generate monodisperse W/O and O/W microemulsions with extremely high controllability. The most popular geometries of droplet generators applied in single emulsion preparation are co-flow, flow-focusing, and T-junction, working under a dripping or jetting regimen [118,137]. The droplet size can be tuned by adjusting the flow rates and ratios of the two phases; however, it is also affected by the viscosity of the dispersed phase, the channel and orifice diameter, and the flow regimes. By a fine tuning of process parameters using low concentrations of nanoparticle precursors, the generation of monodisperse nano-emulsions can be theoretically obtained. Indeed, if the dispersed phase is partially miscible with the continuous one, the formed micro-droplets begin to shrink after the initial creation of an interface leading to the formation of nanoscale systems. Rondeau and Cooper-White [145] fabricated crosslinked alginate-based NGs with an average size ranging from 10 to 300 nm through W/O droplet microfluidics using a low-concentrated aqueous alginate solution and dimethyl carbonate as the dispersed and continuous phase, respectively. Since water and the continuous phase are partially miscible, water diffuses from the polymeric droplets into the transport fluid causing the shrinkage of the drops and the condensation of the polymer phase. However, due to the low concentrations of particle building material and the slow formation of droplets (one by one), the production efficiency of NGs by droplet microfluidics is usually limited and much lower than that of micron-sized particles [137]. 

To address this issue, controlled generation of nanosized mono-dispersed droplets was recently achieved through a novel droplet microfluidics/nanofluidics strategy [146]. Monodisperse nanosized W/O emulsions with controllable sizes were formed from three different protein solutions (i.e., reconstituted silk fibroin, β-lactoglobulin, and lysozyme), which were used as the dispersed phase in a continuous immiscible oil phase (a fluorinated oil containing 2% *w*/*w* fluorosurfactant). These nano-emulsions were then incubated to promote protein self-assembly resulting in the formation of protein nanoscaffolds stabilized by supramolecular fibrils from the three different proteins. The capability of these NGs to penetrate through mammalian cell membrane and deliver their cargo intracellularly was demonstrated, showing their potential as nano-vehicles for drug delivery in biomedical applications. Table 2 enlists the most recent applications of NGs synthesized through microfluidics-assisted technologies.

### 4.2. The Challenge of 3D printing

3D printing represents a milestone in the development of advanced biomaterials for tissue engineering and regenerative medicine approach. In particular, it has revolutionized the traditional design of hydrogel systems improving their properties as excellent cell carriers. This technique has also been implemented for the generation of 3D structured nanogels, where they are 3D-printed in a configuration of drug/photoinitiator-loaded nanoparticles, liposomes, or nano-emulsions suspended in hydrogel matrices [109]. NGs are generally incorporated in hydrogel without affecting the rheological properties of 3D-printed systems and can also carry photoinitiators to improve gelation process or mechanical strength of the printed scaffolds [150]. For example, Liu and coworkers [151] incorporated Pluronic-based NGs encapsulating simvastatin into a 3D-printed porous titanium alloys for orthopedic applications; the combination of them has allowed overcoming the main drawbacks related to the poor compatibility of titanium with bone ingrowth and the limited mechanical properties of NGs, resulting in a composite biomaterial able to promote osteogenesis and neovascularization. Otherwise, NGs intended as building units of a hydrogel network are discussed in the work of Muller et al. [152]: they mixed pure and acrylate-modified Pluronic and, after the printing process and UV exposure, inter-linked nanostructures were formed. By subsequent elution of the unmodified Pluronic, nanostructured hydrogel represented the final product suitable as a bioink and for cell encapsulation.

However, 3D printing of NGs mandates for further research in the academic and industrial sector, and it represents one of the current challenges in the design of these smart nano-vehicles.

## 5. Conclusions

NGs are promising nanosystems to treat a wide range of acute and chronic healthcare scenarios. Different techniques have been developed over the years to meet the criteria of tunable size, morphology, physico-chemical properties, controlled drug delivery and selective cellular uptake. Chemical crosslinking methods, such as polymerization and interfacial reaction, have been increasingly improved to overcome the shortcomings of available formulations with a particular attention to biocompatibility issues. On the other side, physical approaches have been refined to produce NGs showing higher degree of response to biochemical stimuli. Microfluidics and 3D printing represent innovative strategies to produce customized nanoscaffolds for a wide range of applications, thanks to their high efficiency, low cost, and scalability. At present, no conclusive strategy can be identified for NG production: arguably, the smart combination of physico-chemical design principles with advanced manufacturing platforms (e.g., microfluidics and additive manufacturing) will be the driver for innovation in the field of NGs in the near future.

## Figures and Tables

**Table 1:** Controlled/living radical polymerization (CLRP) techniques used in polymerization and NG design.

CLRP Method	Main Features	References
NMP	Suitable for surfactant-free nanoscaffold formation in aqueous dispersion. Reversible termination mechanism between the growing-propagating radicals and the nitroxide, which acts as a control agent. Well-defined structures of the polymeric backbone with the opportunity to add functional moieties before NG formation.	[54,55,56]
ATRP	Fast dynamic equilibrium between radicals and dormant alkyl halides. Catalytic process to activate the dormant alkyl halide, generating a complex with higher oxidation state and a macro-radical ‘living’ polymer. The latter can propagate or is deactivated back to ‘dormant’ polymer, preventing bimolecular termination. Variations vs the traditional use of metal catalysts: activation by electron transfer (ARGET-ATRP), electrochemically mediated transfer (eATRP), zerovalent metals in supplemental activators and reducing agents (SARA-ATRP), initiators for continuous activator regeneration (ICAR-ATRP), photochemical reduction (photoATRP), and metal-free ATRP.	[42,57,58,59,60]
RAFT	Metal-free polymerization chemistry. Use of fast reversible transfer of chain transfer agents (CTA, typically a thio-carbonyl-thio group) to control the propagation of radicals and the formation of dormant species. It ensures a prolonged lifetime of growing chains. Minimal to null undesirable background polymerization.	[61,62,63,64,65]
RITP	Reversible deactivation of radicals mediated by iodine. Synthesis of well-defined polymers with programmable compositions and architectures, narrow molecular weight distributions. Simple reaction system, various types of monomers, mild reaction conditions and tolerance to the reactant purity.	[66,67]
MADIX	The use of the xanthate promotes the design of NGs with high-density of crosslinking points and polymer chains without formation of macrostructures. Possibility of generating core-shell star nanosystems by chain extension from branched polymeric precursors. It can be applied in combination with RAFT.	[53,68,69]

**Table 2:** Microfluidic approaches used in NG design (n.a. = not available drug release studies).

Microfluidic Technique	Materials	Nanogel Size (nm)	Payload	Application Field	References
Microfluidic mixer	Alginate	68–138	Bovine serum albumin (BSA)	Protein delivery	[108]
		45–125	TGF-β3	Growth factors delivery and tissue engineering	[139]
	Hyaluronic acid derivatives	150–400	n.a.	Drug and peptide delivery	[140]
	Hyaluronic acid-cyRGDC derivative	193.2–242.9	Imatinib	Antiangiogenic effect	[141]
	Hyaluronic acid, octenyl and succinic anhydride	115–321	Antimicrobialpeptidomimetic	Antibacterial activity	[147]
		174–194	Anti-biofilm peptide DJK-5	Pseudomonas aeruginosa LESB58 high bacterial density infection	[148]
	2,2’(ethylenedioxy)diethanethiol (EDDET)	60–70	Rhodamine B	Tumor therapy	[142]
	Alginate	10–300	n.a.	Multipurpose	[145]
Droplet generator	Proteins (reconstituted silk fibroin, β-lactoglobulin, lysozyme)	50–2500	Fluorescent marker	Intracellular delivery	[146]
	Hyaluronic acid	80–160	Proteins	Cancer therapy	[149]
